# Sensory nerve transfers in the upper limb after peripheral nerve injury: a scoping review

**DOI:** 10.1177/17531934231205546

**Published:** 2023-11-21

**Authors:** Liron S. Duraku, Caroline A. Hundepool, Amy M. Moore, Kyle R. Eberlin, J. Michiel Zuidam, Samuel George, Dominic M. Power

**Affiliations:** 1 The Hand & Peripheral Nerve Injury Department, University Hospitals Birmingham NHS Foundation Trust, Birmingham, UK; 2 Department of Plastic, Reconstructive & Hand Surgery, Amsterdam UMC, Amsterdam, the Netherlands; 3 Department of Plastic, Reconstructive & Hand Surgery, Erasmus MC, Rotterdam, the Netherlands; 4 Department of Plastic and Reconstructive Surgery, The Ohio State University Wexner Medical Center, Columbus, OH, USA; 5 Division of Plastic and Reconstructive Surgery, Massachusetts General Hospital, Harvard Medical School, Boston, MA, USA

**Keywords:** Nerve transfer, peripheral nerve, upper limb

## Abstract

Nerve transfer for motor nerve paralysis is an established technique for treating complex nerve injuries. However, nerve transfer for sensory reconstruction has not been widely used, and published research on this topic is limited compared to motor nerve transfer. The indications and outcomes of nerve transfer for the restoration of sensory function remain unproven. This scoping review examines the indications, outcomes and complications of sensory nerve transfer. In total, 22 studies were included; the major finding is that distal sensory nerve transfers are more successful than proximal ones in succeeding protective sensation. Although the risk of extension of the sensory deficit with donor site loss and morbidity from neuromas remain a barrier to wider adoption, these complications were not reported in the review. Further, the scarcity of studies and small patient series limit the ability to determine sensory nerve transfer success. However, sensory restoration remains an opportunity for surgeons to pursue.

**Level of evidence:** II

## Introduction

Sensory perception is critical to normal interaction with our environment (Graczyk et al., 2019). Nociceptive function warns us of injury, prompts withdrawal from a noxious stimulus and prevents further tissue damage. For the hand, sensation provides tactile gnosis enabling discrimination of texture and shape. Pressure and vibration detection provide further modalities for interaction with our environment, and proprioceptive function enables precise positioning with fine control. Diminished sensation after peripheral nerve injury or in the setting of peripheral neuropathy risks recurrent injury to the trophic skin (Menorca et al., 2013). Restoration of sensation is a key objective in the management of a peripheral nerve injury; however, it has received limited attention when compared to the restoration of motor function.

Nerve reconstructions after proximal lesions with long reinnervation distances, large nerve gaps and a poor surgical bed are associated with inferior outcomes (Moore et al., 2015). Nerve transfers are helpful when it is not possible to anatomically reconstruct the primary neurological lesion or when unfavourable factors, such as increased length of regeneration distance from injury to the target organ, may limit the effectiveness of such repairs. Nerve transfer surgery involves cutting an expendable donor nerve and performing a tension-free coaptation to the sectioned distal nerve stump of a more critical yet non-functioning target (Moore et al., 2015). Expandable donor nerve is usually in the context of a motor nerve transfer with very little donor morbidity, such as weakness or paralysis.

In the context of a sensory nerve transfer, this includes widening the area of sensory loss in the expectation of some recovery to a critical target zone. In addition, there is an inherent risk of sensory transfers that includes creating a symptomatic neuroma at the site of the donor nerve causing neuropathic pain and further limiting the function of the extremity. These may be the reasons why nerve transfers for sensory reconstruction have still not been widely adopted by hand surgeons.

Most reported sensory nerve transfers are small case series with variable results and limited evidence, which limits the wider adoption of the technique. After a life-changing nerve injury, discussing further sensory sacrifice with a patient regarding potential improvements of sensation in more critical areas without certainty of outcome remains contentious, especially when there is a risk of triggering or worsening neuropathic pain.

At present, the indications for sensory nerve transfer remain unclear and the reliability of outcomes is unknown. Research into sensory nerve transfer should focus on quantitative sensory recovery, functional improvement, the effect on neuropathic pain mitigation and provide data on complications of this intervention, including donor sensory loss and risk of precipitation of neuropathic pain. The aim of this scoping review was to collect all available evidence for sensory nerve transfer surgery, including indications, outcomes and complications, and provide guidance on when this technique may be indicated.

## Methods

### Literature search

We conducted a systematic review of nerve transfer for sensory restoration in the upper extremity according to the Preferred Reporting Items for Systematic Reviews and Meta-Analyses statement (PRISMA) guidelines in the Embase, MEDLINE, Web of Science and Cochrane Central databases (Online supplementary material S1).

The inclusion criteria and search terms were formulated with the aid of a medical librarian. The full search terms can be found in the online supplementary material. Two authors (LD and CH) independently selected studies that met the inclusion criteria on the basis of the title and abstract. Studies on cadavers and anatomical studies were excluded, as were animal studies, reviews, conference reports, non-English studies and studies for which full text was not available. Agreement on inclusion was discussed in consensus meetings.

The same two authors (LD and CH) extracted the year of publication, study method, number of patients included, type of nerve transfer, time from injury to surgery and time to follow-up using a standardized data collection form. Outcome measures differed between studies, so any outcome on sensory recovery was collected. The strength of evidence of all studies was assessed using the Jovell and Narvarro-Rubio classification (Table S2) (Jovell and Navarro-Rubio, 1995).

### Statistical analysis

The primary outcome measurement of this systematic review was the Zachery and Highet scale, with a score of S3 or higher considered a good clinical outcome. The proportion of patients reaching this score was calculated as a percentage. Because of the heterogeneity of the patient cohort, small sample size and variation in reporting, conducting a statistical analysis was not possible.

## Results

**Figure 1.**
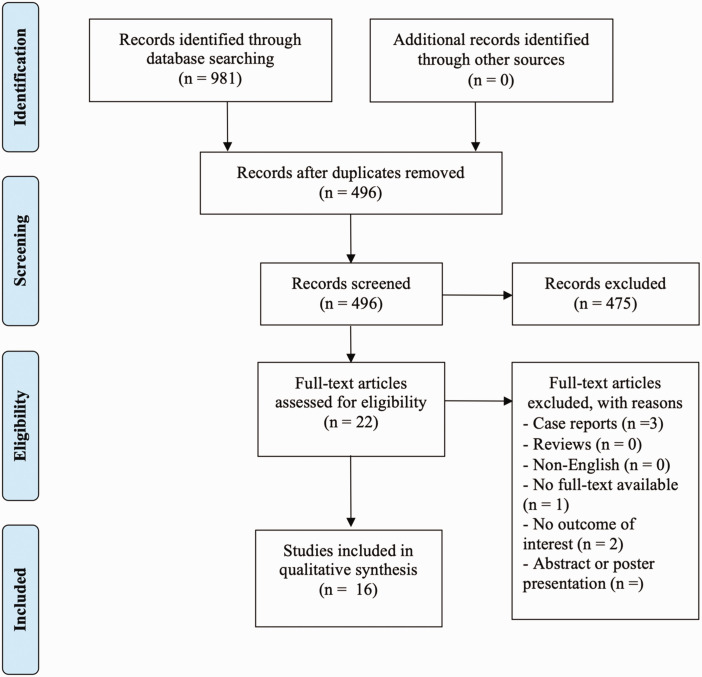
Flow chart regarding the selection of included articles.

The initial literature search identified 981 publications. After the removal of duplicates, 496 studies were screened based on the title and abstract. A total of 22 studies were included for full text screening. In total, 16 studies finally met the inclusion criteria and were included in this scoping review (Figure 1). Most included studies were case reports and case series, with just a few cohort or prospective studies.

The overview on the included studies is depicted in Table 1. Three studies investigated outcomes after pan-brachial plexus injuries and three lower brachial plexus injuries. Seven studies investigated the ulnar nerve, four reported on the median nerve and three looked at digital nerve injuries. The outcome of the different nerve transfers is depicted in Table 2. The studies are sorted per nerve injury for which different nerve transfers are used.

**Table 1.** Included studies in this scoping review.

Author, year	Level of evidence^a^	Study design	No. of patients	Type of nerve injury	Outcome measurement
Matloubi, 1988	6	Retrospective cohort	25	Ulnar and median	2PD
Stocks et al., 1991	6	Retrospective cohort	17	Median, ulnar, digital	s2PD, m2PD
Ihara et al., 1996	8	Case report	15	Pan-BPI	m2PD, SW
Battiston and Lanzetta, 1999	8	Case report	7	High ulnar (above elbow)	s2PD, m2PD
Ozkan et al., 2001	6	Prospective cohort	20	Ulnar + median + BPI (C5/C6)	s2PD, m2PD, SW
Voche and Ouattara, 2005	8	Case report	10	Digital nerve	s2PD, m2PD
Hattori et al., 2009	7	Retrospective cohort	17	Pan-BPI	s2PD, m2PD, SW
Bertelli and Ghizoni, 2011	8	Case report	8	Median proximal elbow	SW
Leechavengvongs et al., 2011	8	Case report	8	C5/C6 BPI	SW
Flores, 2011	8	Case report	5	Ulnar proximal	s2PD, SW
Bertelli et al., 2012	6	Retrospective cohort	8	Lower BPI	s2PD, SW
Chen et al., 2013	6	Retrospective cohort	17	Proper digital nerve	s2PD, SW
Flores, 2015	6	Retrospective cohort	20	Ulnar (proximal)	s2PD, SW
Sallam et al., 2017	6	Retrospective cohort	24	Ulnar (proximal)	s2PD
Foroni et al., 2017	8	Case report	11	Pan-BPI	s2PD, m2PD, SW
Emamhadi and Andalib, 2018	8	Case report	6	C5/C6 BPI	s2PD

a Level of evidence according to the classification by Jovell and Navarro-Rubio (1995) (Table S2).

BPI: brachial plexus injury; m2PD: moving two-point discrimination; s2PD: static two-point discrimination; SW: Semmes–Weinstein testing.

**Table 2.** Clinical outcome of sensory nerve transfer based on nerve injury.

Author, year	Type of nerve transfer	No. of patients	Time from trauma to surgery (months)	Time to follow-up (months)	Highet and Zachery scale (≥S3)
*Pan-BPI*					
Ihara et al., 1996	ICN III–IV lateral cutaneous branch → median nerve; ete	3	(24–72)	48	0/3 (0)
	SCN → lateral cord median nerve; ete	7	(1–14)	34	0/7 (0)
Hattori et al., 2009	ICN → median; ete	14	(3–8 years)	4.1 years	0/14 (0)
	ICN → ulnar; ete	3	(3–22)	10	0/3 (0)
Foroni et al., 2017	Intercostal brachial → med; ete	11	7 (2–11)	41 (36–52)	2/11 (18)
*C5/6 – BPI*					
Leechavengvongs et al., 2011	SRN → med; ste	8	8 (3–18)	28 (24–36)	2/8 (25)
Emamhadi and Andalib, 2018	Ulnar fourth webspace → first webspace (median); ete	6	7 (5–10)	34 (30–36)	6/6 (100)
Bertelli et al., 2012	PBMN → ulnar (proper digital little finger); ete	8	8 (3–28)	24	8/8 (100)
Ozkan et al., 2001	Digital nerve transfer ulnar → first webspace; ete	6	13 (0–60)	78 (48–119)	6/6 (100)
	Digital nerve transfer median → lateral border; ete	6			6/6 (100)
	RSN → first webspace; ete	1			1/1 (100)
*Ulnar nerve injury*					
Matloubi, 1988	RSN → ulnar; ete	7	(6–36)	(19–58)	4/7 (57)
Battiston and Lanzetta, 1999	PBMN → sensory branch ulnar nerve; ete	7	4 (1–5)	2.5 years	7/7 (100)
Flores, 2015	Sensory ulnar nerve → third common digital nerve; ets	15	7	24	6/15 (40)
Sallam et al., 2017	Third webspace → sensory superficial branch ulnar nerve; ete + dorsal sensory ulnar → median nerve; ets	24	9 (6–18)	29 (24–38)	14/24 (58)
Flores, 2011	Sensory ulnar nerve → third common digital nerve; ets	5	7 (4–10)	20 (15–30)	5/5 (100)
*Median nerve injury*					
Bertelli and Ghizoni, 2011	Dorsal RSN → proper digital first web; ete	8	7 (3–12)	12	8/8 (100)
Matloubi, 1988	RSN → median; ete	18	(6–36)	(19–58)	8/12 (66)
*Mixed nerve injury*					
Stocks et al., 1991	Proper non-critical digital nerve transfer to critical digit; ete	12	<6	78 (301–304)	10/12 (85)
*Digital nerve injury*					
Voche and Ouattara, 2005	Digital nerve → uninjured digital nerve; ets	10	<1 day	16 (9–29)	10/10 (100)
Chen et al., 2013	Dorsal digital branch → proper digital nerve; ete	21	<1 day	25	21/21 (100)

Data are presented as n (%) or mean (range).

BPI: brachial plexus injury; ete: end-to-end anastomosis; ets: end-to-side anastomosis; ICN: intercostal nerve; PBMN: palmar branch median nerve; RSN: radial sensory nerve; SCN: supraclavicular nerve.

### Brachial plexus injuries

For the pan-brachial plexus injuries, three studies reported the outcome of proximal nerve transfers using an intercostal or supraclavicular (C4) nerve transfer either to the median or ulnar nerve (Foroni et al., 2017; Hattori et al., 2009; Ihara et al., 1996). Although these articles described the return of some protective sensibility, most patients did not achieve S3 or higher sensory recovery. Only the intercostobrachial nerve to median nerve transfer, described by Foroni et. al. (2017) showed 2 (18%) patients receiving a score of S3+.

In the lower brachial plexus injury, distal sensory nerve transfers were described in four studies. The sensory branch of the radial nerve (Leechavengvongs et al., 2011), the palmar branch of the median nerve (Bertelli and Ghizoni, 2011), the fourth webspace (Emamhadi and Andalib, 2018) or other digital nerve transfers (Ozkan et al., 2001) were used as donors for transfer in a total of 35 patients. A score of S3 or higher was obtained in 25%–100% of patients in those studies.

### Other peripheral nerve injuries

Ulnar nerve injuries were treated with end-to-end sensory nerve transfers from the sensory branch of the radial nerve (Matloubi, 1988), the palmar branch of the median nerve (Battiston and Lanzetta, 1999), a reverse end-to-side to the third webspace common digital nerve (Flores, 2011, 2015) or third webspace end-to-side to the sensory ulnar nerve (Sallam et al., 2017). There was a total of 58 patients and there was no clear distinction in outcome between the different types of sensory transfer reconstruction. A score of S3 or higher was obtained in 40%–100% of patients.

A total of 26 patients with median nerve injuries received a sensory radial nerve transfer end-to-end in two studies (Bertelli and Ghizoni, 2011; Matloubi, 1988). These distal nerve transfers showed a good sensory recovery (S3 or greater) in 66%–100% of the patients.

### Digital nerve injuries

Stocks et al. (1991) described non-critical digital nerve end-to-end transfers to critical digits in both ulnar, median and digital nerve injuries and reported a good outcome in 10 out of 12 patients. Two papers with a total of 31 patients investigated digital nerve transfers (incorporating both end-to end and end-to-side techniques) for digital nerve injuries (Chen et al., 2013; Voche and Ouattara, 2005). All transfers were performed on the same day as the trauma and all had a good sensory outcome (>S3: 100%); there was no difference between the end-to-end and end-to-side technique.

There is vast heterogeneity in the reporting of results, with two-point discriminations (static or movement) and monofilament examination; therefore, it was not possible to compare these results across the studies.

No articles reported complications of neuropathic pain or complaints of donor site morbidity. Only one study reported cold intolerance in three out of 10 patients after sensory nerve transfers in the digits. This was assessed by a simple yes or no answer (Voche and Ouattara, 2005). Some studies where patients underwent sensory nerve transfers reported in the original period (3–6 months) some sensory crossed innervation, which is the perceived sensation of the donor area on the recipient area. The sensory crossed innervation phenomena were transient in all patients.

## Discussion

The current scoping review shows that sensory nerve transfers can potentially restore protective sensation to critical areas of the hand after peripheral nerve injuries, especially in distal nerve transfers but less so in proximal ones. Most of the studies are small case series; however, it seems that many of the sensory transfers that are performed in the lower arm and hand show reliable protective recovery at the S3 level or greater. Conversely, the more proximal sensory transfers in pan-plexus injuries seldom achieve S3. In addition, it is interesting to note that the current systematic review only identified 22 studies for inclusion (excluding case reports), just a fraction of the number of studies reported for motor nerve transfer surgery.

The first successful sensory nerve transfer in 1921 described transfer of the superficial radial nerve to the median nerve after a segmental loss, restoring some feeling to the hand (Harris, 1921). The critical sensory area of the hand is viewed as the opposing pulps of the index and thumb innervated by the median nerve through the radial and ulnar digital nerves, respectively, and the ulnar aspect of the hand and small finger innervated by the ulnar nerve. Reconstruction options for loss of median sensation in the first web can include transfer of digital nerves innervated through the ulnar nerve from the fourth web. These may be coapted as end-to-end nerve transfers in the palm of the hand. Conversely, the third webspace sensory innervation from the median nerve may be used to restore sensation to the ulnar side of the hand. Using end-to-side nerve transfer, it may be possible to restore some sensory function without complete loss of the donor sensory function; however, there are no large-scale series to report on the quality of the sensory recovery when employing these advanced techniques. Most articles used an end-to-end technique for the sensory nerve transfer, demonstrating a predominance of S3 outcomes in the distally sited transfers. The small numbers of successful end-to-side transfers warrants further exploration; however, the variation in reported end-to-side techniques proposes a challenge and the method of exposure of the donor nerve and the depth of inset of the recipient require further investigation to optimize this technique and ensure reproducible results and to standardize terminology when reporting.

The importance of protective sensation is demonstrated by the morbidity of sensory neuropathy with poor skin condition, risk of trophic ulceration and secondary complications, including osteomyelitis and amputation (Axelrod and Gold-von Simson, 2007). The minimal sensory recovery standard at S3 proposed in the Highet and Zachary scale (Wang et al., 2013; Zachary and Holmes, 1946) may be sufficient to provide some cutaneous protection, but the level required for useful functional gains is ill-defined and likely to vary depending on the site and nerve involved. The potential functional gains should form part of future research areas and should include both subjective and objective measures of sensory function as well as validated patient-reported outcome measures. These studies should also examine the relative importance of perceived critical and non-critical cutaneous innervation.

The principle of sensory nerve transfer for the restoration of sensation to critical areas of the hand through sacrifice of sensation in less critical areas is elegant, but it is not without morbidity. The indications for sensory transfer will evolve as the evidence base matures. Currently, decisions for transfer are largely based on the surgeon’s preference and experience, remaining in the domain of the specialist peripheral nerve surgeon. The ability for sensory end-organ reinnervation beyond the 12 months, commonly defined as a cut-off for successful motor reinnervation, provides the surgeon with an opportunity to use sensory nerve transfer as a secondary salvage procedure when primary anatomical nerve repair has failed and there are trophic complications in the denervated skin. All included articles in this systematic review had sensory reconstructions within 12 months after trauma. The review has highlighted that sensory recovery is superior in distal sensory nerve transfers when compared with proximal transfers after pan-plexus injury where few cases achieve S3 recovery. The reasons are likely to be multifactorial and may include the longer reinnervation distances, the severity of injury, regenerating axon spread to larger cutaneous fields, the quality of the donor nerve, and the inevitable apoptosis in the dorsal root ganglion that follows longstanding and proximal peripheral nerve injuries (Terenghi et al., 2011).

Conversely, two studies that investigated nerve grafting and distal sensory nerve transfer in proximal ulnar nerve lesions showed similar sensory recovery in most cases at S3 (Flores, 2015; Sallam et al., 2017). As could be predicted by the shorter reinnervation distance and use of an undamaged healthy donor cell population, one of the studies found that the sensory transfer group had faster sensory recovery compared to the nerve graft group (Sallam et al., 2017). It remains unclear how time-sensitive sensory reinnervation really is. Timing in motor nerve transfer is crucial for good outcomes as motor endplate viability is dependent on time. However, in sensory reconstruction, it is debated that the sensory end organs are more resistant to apoptosis than the motor endplates. However, this is an assumption that has not been investigated in humans.

In all of the included studies, there were no clear indications for reconstruction other than the absence of sensation in the target area. However, in severely neglected carpal tunnel syndrome when poor sensation leads to clumsiness, sensory nerve transfer surgery is not offered. One might argue that poor sensation rather than absent sensation leads to a more limited functional deficit. The ability of sensory nerve transfers to provide useful recovery at the S3 level provides a further opportunity for research; however, the effect of chronic denervation of the cutaneous sensory end organs is not fully known and may preclude useful uplift of function. Only one study reported cold intolerance in three out of 10 patients after sensory nerve transfers in the digits (Voche and Ouattara, 2005). However, it was not clear if the cold intolerance was due to the initial trauma or due to the digit nerve transfer. None of the other included studies reported donor-site related morbidities or complications such as neuroma incidence or prevalence of neuropathic pain.

**Figure 2.**
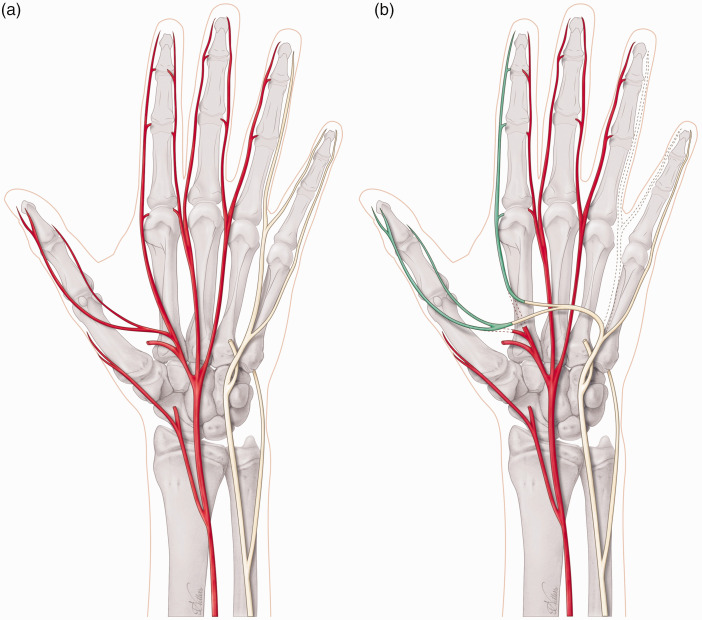
(a) Non-functioning (red) median nerve and functioning (yellow) ulnar nerve and (b) Sensory nerve transfer of fourth webspace (dotted, ulnar nerve) to the first webspace (green, median nerve).

**Figure 3.**
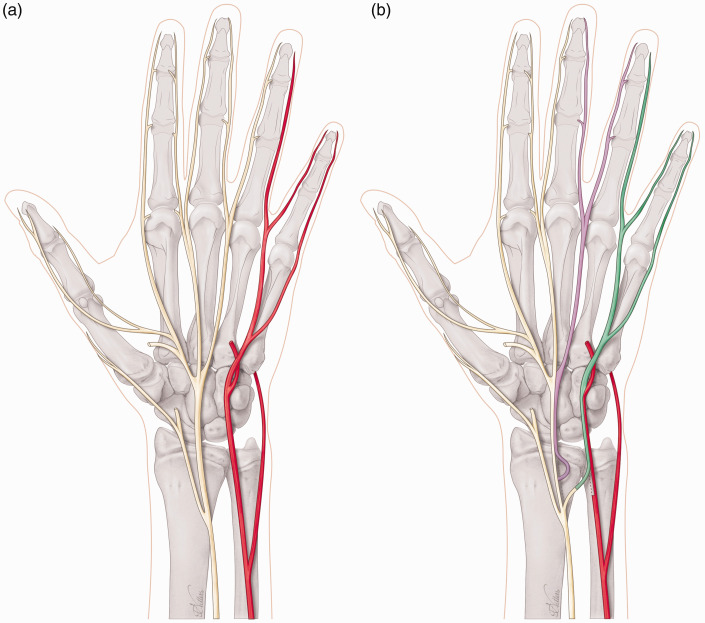
(a) Non-functioning (red) ulnar nerve and functioning (yellow) median nerve and (b) Sensory nerve transfer of third webspace (purple, median nerve) to the sensory part of ulnar nerve (green) and end-to-side coaptation of proximal end of donor third webspace to main median nerve for donor site reinnervation.

**Figure 4.**
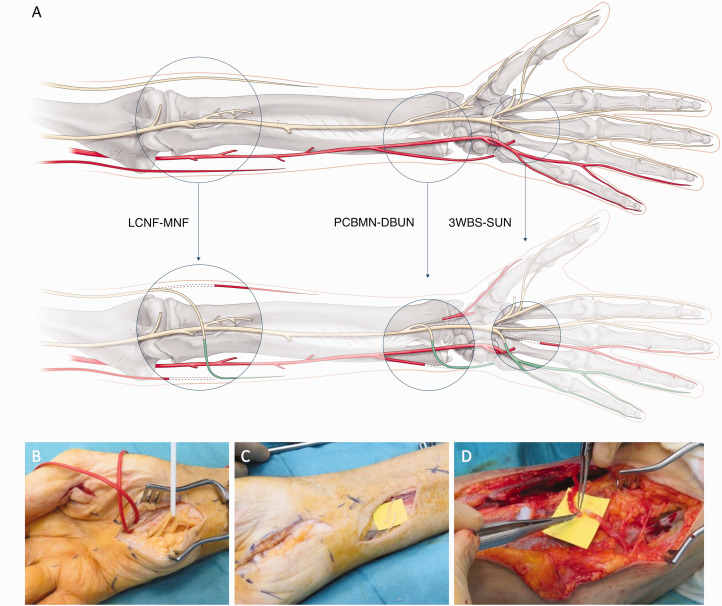
(a) Example of a complex sensory nerve transfer combination. A 71-year-old man with en-bloc excision of a chondrosarcoma that included sacrifice of the right C8 and T1 nerve roots. For sensory reconstruction of the ulnar side of the arm, three transfer were performed. Sensory nerve transfers included a 3WBS-C7 to the SUN for sensation to the ulnar border of the small finger. The PCBMN was transferred to the DBUN for sensation to the ulnar border of the hand. The LCNF was transferred to the MCNF for sensation restoration to the ulnar border of the forearm. Follow-up at 18 months demonstrated that the patient had regained protective sensation (S3+) to the ulnar side of the small finger, the dorso-ulnar hand and the medial forearm. (b) Sensory nerve transfers included a 3WBS-C7 (red loop) to the SUN (white loop). (c) PCBMN transfer to the DBUN. (d) LCNF transfer to the MCNF. Orientation forearm: right-proximal and left-distal. 3WBS-C7: third webspace common digital nerve transfer; DBUN: dorsal branch of the ulnar nerve; LCNF: lateral cutaneous nerve of the forearm; MCNF, medial cutaneous nerve of the forearm; PCBMN: palmar cutaneous branch of the median nerve; SUN: superficial ulnar nerve.

To restore the first webspace sensation after a high median nerve injury or a C6 root injury, it is possible to use the third or fourth webspace nerve fascicles that can be transferred in the palm or at the distal edge of the carpal tunnel (Figure 2). The same reconstruction could be offered in combined C5 and C6 brachial plexus injuries; however, in our experience, we seldom encounter patients who are troubled by the sensory loss of an upper brachial plexus injury. Perhaps the most impactful loss is motor, and sensory dysfunction is generally tolerated well in most patients. Another more commonly sensory nerve transfer is after a high ulnar nerve injury, in which the third webspace can be transferred just at the level of the carpal tunnel to the sensory part of the ulnar nerve to provide sensation to the small finger and ulnar aspect of the hand (Figure 3). Other less commonly used nerve transfers are from the lateral antebrachial cutaneous nerve (LABCN) to the radial nerve after injury, terminal superficial radial nerve to first webspace digital nerves in mid-cervical tetraplegia and transfer of the palmar branch of the median nerve to the dorsal branch of the ulnar nerve for complex proximal loss of the ulnar nerve. In isolated upper brachial plexus surgery reconstruction, the sensory branches of the axillary nerve may be transferred end-to-side to the radial nerve, and for pan-plexus injuries where trophic ulceration of the insensate hand is common, nerves may be transferred to the median nerve for sensory restoration to the radial hand using sensory intercostals, supraclavicular nerves or the contralateral C7 root. In addition, it is possible to utilize a combination of these reported sensory nerve transfers to reconstruct complex nerve injuries (Figure 4). For reconstruction of composite tissue loss after trauma or oncological resection, sensory neurotization of free flaps involving cutaneous tissue may be useful in providing protective sensation and reducing neuropathic pain from otherwise redundant sensory nerve branches without distal targets (Rinkinen et al., 2020).

When compared to grafting, a disadvantage of sensory nerve transfer is that it may not address the possible neuropathic pain that can accompany a proximal nerve injury. Anatomical restoration of nerve continuity with afferent signalling is thought to have a positive impact on the pain that follows an injury. However, nerve grafting has its own set of limitations, as outcomes may be affected by age, location of injury, specific affected nerve, time from injury, gap length and mechanism of injury. In cases with multiple poor prognostic factors for nerve grafts, nerve transfers may have a theoretical advantage. In study by Sallam et al. (2017), they avoided grafts longer than 5 cm, because of anticipated inferior outcome and favoured sensory nerve transfer.

A limitation of the current literature is that the majority of the studies are small descriptive case series. In addition, there is significant heterogeneity in how sensation is measured and what kind of outcomes are used. Hooper and Ruettermann (2022) advised a few guidelines to report numeric values in sensory testing with movement and static two-point discrimination and Semmes–Weinstein testing: (1) who performed the test to identify possible observer bias; (2) at what time point was the test performed in the follow-up; and (3) if the results were uniformly reported. In the future, adoption of these guidelines would enable more meaningful comparisons between different studies.

In summary, this scoping review found that protective sensation is possible with nerve transfers, and distal nerve transfers appear to be more successful than more proximal ones. Further, the scarcity of studies and small patient series limit the ability to determine the success of sensory nerve transfers. However, sensory restoration is an opportunity for surgeons to pursue; however, the authors recognize further studies are needed to prove efficacy. Although the risk of extension of the sensory deficit with donor site loss and morbidity from neuromas may be a barrier to wider adoption, we did not find these complications to be reported in the studies of this review. The role of end-to-side transfer to minimize donor site morbidity has promise theoretically but is not yet substantiated in the literature. Further exploration of these issues is required before any meaningful conclusions regarding sensory nerve transfer may be made.

## Supplemental Material

sj-pdf-1-jhs-10.1177_17531934231205546 - Supplemental material for Sensory nerve transfers in the upper limb after peripheral nerve injury: a scoping review Supplemental material, sj-pdf-1-jhs-10.1177_17531934231205546 for Sensory nerve transfers in the upper limb after peripheral nerve injury: a scoping review by Liron S. Duraku, Caroline A. Hundepool, Amy M. Moore, Kyle R. Eberlin, J. Michiel Zuidam, Samuel George and Dominic M. Power in Journal of Hand Surgery (European Volume)

sj-pdf-2-jhs-10.1177_17531934231205546 - Supplemental material for Sensory nerve transfers in the upper limb after peripheral nerve injury: a scoping review Supplemental material, sj-pdf-2-jhs-10.1177_17531934231205546 for Sensory nerve transfers in the upper limb after peripheral nerve injury: a scoping review by Liron S. Duraku, Caroline A. Hundepool, Amy M. Moore, Kyle R. Eberlin, J. Michiel Zuidam, Samuel George and Dominic M. Power in Journal of Hand Surgery (European Volume)
