# Supplementary material for: Sensory nerve transfers in the upper limb after peripheral nerve injury: a scoping review
Source: J Hand Surg Eur Vol. 2023 Nov 21;49(8):946–55. doi: 10.1177/17531934231205546 (PMC11382435; doi:10.1177/17531934231205546)
Supplement: sj-pdf-1-jhs-10.1177_17531934231205546 - Supplemental material for Sensory nerve transfers in the upper limb after peripheral nerve injury: a scoping review [file sj-pdf-1-jhs-10.1177_17531934231205546.pdf]

Appendix A: Search terms

Embase.com 273

('sensory nerve transfer'/de OR ((Nerve NEAR/6 (transfer*))):ab,ti OR (transfer* AND nerve*):ti) AND (sensibility/de OR 'sensory system'/exp OR 'sensory nerve transfer'/de OR (sensibilit* OR sensor* OR somatosensor*):ab,ti) AND (limb/exp OR 'limb injury'/exp OR 'radial nerve'/exp OR 'brachial plexus'/exp OR 'brachial plexus injury'/exp OR 'femoral nerve'/de OR 'peroneus nerve'/de OR 'tibial nerve'/de OR 'saphenous nerve'/de OR 'sural nerve'/de OR 'sciatic nerve'/de OR (extremit* OR limb* OR arm OR leg OR arms OR legs OR hand* OR wrist OR finger* OR thumb* OR foot OR feet OR heel OR ((axillary OR median OR musculocutaneous OR radial OR ulnar OR femoral OR peroneus OR tibial OR saphenous OR sural OR sciatic) NEAR/3 nerve*) OR (brachial* NEAR/3 plexus)):ab,ti) NOT ([animals]/lim NOT [humans]/lim) NOT [conference abstract]/lim AND [english]/lim

Medline ALL Ovid 344

(Nerve Transfer / OR ((Nerve ADJ6 (transfer*))).ab,ti. OR (transfer* AND nerve*).ti.) AND (exp Sense Organs / OR (sensibilit* OR sensor* OR somatosensor*).ab,ti.) AND (exp Extremities / OR Radial Nerve / OR Brachial Plexus / OR Femoral Nerve / OR Tibial Nerve / OR Sural Nerve / OR Sciatic Nerve / OR (extremit* OR limb* OR arm OR leg OR arms OR legs OR hand* OR wrist OR finger* OR thumb* OR foot OR feet OR heel OR ((axillary OR median OR musculocutaneous OR radial OR ulnar OR femoral OR peroneus OR tibial OR saphenous OR sural OR sciatic) ADJ3 nerve*) OR (brachial* ADJ3 plexus)).ab,ti.) NOT (exp animals/ NOT humans/) AND english.la.

Web of Science 301

TS=((((Nerve NEAR/5 (transfer*))) OR (transfer* AND nerve*):ti) AND ((sensibilit* OR sensor* OR somatosensor*)) AND ((extremit* OR limb* OR arm OR leg OR arms OR legs OR hand* OR wrist OR finger* OR thumb* OR foot OR feet OR heel OR ((axillary OR median OR musculocutaneous OR radial OR ulnar OR femoral OR peroneus OR tibial OR saphenous OR sural OR sciatic) NEAR/2 nerve*) OR (brachial* NEAR/2 plexus)))) NOT DT=(Meeting Abstract OR Meeting Summary) AND LA=(english)

Cochrane 63

(((Nerve NEAR/6 (transfer*))):ab,ti OR (transfer* AND nerve*):ti) AND ((sensibilit* OR sensor* OR somatosensor*):ab,ti) AND ((extremit* OR limb* OR arm OR leg OR arms OR legs OR hand* OR wrist OR finger* OR thumb* OR foot OR feet OR heel OR ((axillary OR

median OR musculocutaneous OR radial OR ulnar OR femoral OR peroneus OR tibial OR saphenous OR sural OR sciatic) NEAR/3 nerve*) OR (brachial* NEAR/3 plexus)):ab,ti)
