# Supplementary material for: Sensory nerve transfers in the upper limb after peripheral nerve injury: a scoping review
Source: J Hand Surg Eur Vol. 2023 Nov 21;49(8):946–55. doi: 10.1177/17531934231205546 (PMC11382435; doi:10.1177/17531934231205546)
Supplement: sj-pdf-2-jhs-10.1177_17531934231205546 - Supplemental material for Sensory nerve transfers in the upper limb after peripheral nerve injury: a scoping review [file sj-pdf-2-jhs-10.1177_17531934231205546.pdf]

Supplementary Table 1. Classification of strength of evidence by Jovell and Narvarro-Rubio

Level	Strenght of Evidence	Type of study design
1	Good	Meta-analysis of randomized controlled trials
2		Large-sample randomized controlled trials (N>25 for each group)
3	Good to fair	Small-sample randomized controlled trials (N<25 for each group)
4		Non-randomized controlled prospective trials
5		Non-randomized controlled retrospective trials
6	Fair	Cohort studies
7		Case-control studies
8	Poor	Noncontrolled clinical series; descriptive studies
9		Anecdotes or case reports
